# Cyclophosphamide-Induced Cystitis Increases Bladder CXCR4 Expression and CXCR4-Macrophage Migration Inhibitory Factor Association

**DOI:** 10.1371/journal.pone.0003898

**Published:** 2008-12-10

**Authors:** Pedro L. Vera, Kenneth A. Iczkowski, Xihai Wang, Katherine L. Meyer-Siegler

**Affiliations:** 1 Bay Pines VA Healthcare System, Research & Development (151), Bay Pines, Florida, United States of America; 2 Department of Surgery, College of Medicine, University of South Florida, Tampa, Florida, United States of America; 3 Prostate Cancer Research Laboratories, University of Colorado-Denver, Aurora, Colorado, United States of America; 4 Department of Molecular Medicine, College of Medicine, University of South Florida, Tampa, Florida, United States of America; Instituto Oswaldo Cruz and FIOCRUZ, Brazil

## Abstract

**Background:**

Macrophage migration inhibitory factor (MIF) is a pro-inflammatory cytokine involved in cystitis and a non-cognate ligand of the chemokine receptor CXCR4 in vitro. We studied whether CXCR4-MIF associations occur in rat bladder and the effect of experimental cystitis.

**Methods and Findings:**

Twenty male rats received saline or cyclophosphamide (40 mg/kg; i.p.; every 3^rd^ day) to induce persistent cystitis. After eight days, urine was collected and bladders excised under anesthesia. Bladder CXCR4 and CXCR4-MIF co-localization were examined with immunhistochemistry. ELISA determined MIF and stromal derived factor-1 (SDF-1; cognate ligand for CXCR4) levels. Bladder CXCR4 expression (real-time RTC-PCR) and protein levels (Western blotting) were examined. Co-immunoprecipitations studied MIF-CXCR4 associations.Urothelial basal and intermediate (but not superficial) cells in saline-treated rats contained CXCR4, co-localized with MIF. Cyclophosphamide treatment caused: 1) significant redistribution of CXCR4 immunostaining to all urothelial layers (especially apical surface of superficial cells) and increased bladder CXCR4 expression; 2) increased urine MIF with decreased bladder MIF; 3) increased bladder SDF-1; 4) increased CXCR4-MIF associations.

**Conclusions:**

These data demonstrate CXCR4-MIF associations occur in vivo in rat bladder and increase in experimental cystitis. Thus, CXCR4 represents an alternative pathway for MIF-mediated signal transduction during bladder inflammation. In the bladder, MIF may compete with SDF-1 (cognate ligand) to activate signal transduction mediated by CXCR4.

## Introduction

Macrophage migration inhibitory factor (MIF) is an ubiquitous pleiotropic cytokine involved in cell proliferation and inflammation [1], [2]. MIF plays an important and unique role in inflammation since MIF stands upstream of other pro-inflammatory mediators and it can counter-regulate the anti-inflammatory effects of glucocorticoids [2]. MIF is implicated in animal models of inflammatory diseases, including arthritis, glomerulonephritis, acute lung injury and sepsis (for recent review [3]).

Our recent experimental evidence indicates that MIF participates in bladder inflammation since: (1) MIF is constitutively expressed in the urothelium [4], [5]; (2) bladder MIF expression is upregulated in different models of experimental cystitis in animals [6], [7]; (3) MIF is released from the bladder during experimental cystitis [6], [8], [9] and urinary tract infections in humans [10] and finally, (4) neutralizing MIF with intravesical antibodies decreased experimental bladder inflammation [7]. Thus, based on our experimental observations, our hypothesis of a pro-inflammatory role for MIF during bladder inflammation agrees well with MIF's pro-inflammatory role in several disease models (e.g. arthritis, Crohn's disease) where treatment with neutralizing MIF antibodies results in decreased inflammation [11], [12].

The mechanism for MIF's action is not completely defined and remains an active area of investigation. MIF may exert autocrine effects through binding to intracellular JAB1 [13] and also paracrine effects by binding to cell-surface receptors [14]. Until recently, complex formation between MIF and cell-surface CD74 was the only described mechanism for MIF-receptor interaction [15] . CD74 is part of the major histocompatibility class-II (MHC-II) complex; however, a small amount of CD74 can be found on the cell-surface not associated with MHC-II [16]. MIF binds to cell-surface CD74 [15] and the MIF-CD74 complex then activates signal transduction by binding to another cell-surface receptor, CD44 [14]. We showed that MIF, CD44 and CD74 are all upregulated in the urothelium after experimental inflammation in rats [6], [17]. Therefore, all of the components are in place during bladder inflammation for MIF-activated signal transduction to occur.

Recently, however, a novel functional association between MIF and chemokine receptors CXCR2 and CXCR4 was described in T cells in vitro [18]. Chemokines are small proteins that direct leukocyte traffic to sites of inflammation or injury [19]. CXCR4 is a G-protein coupled receptor for stromal cell-derived factor-1 (SDF-1/CXCL12). Although chemokines typically display a high degree of receptor promiscuity, CXCR4 was (until recently) thought to bind only to SDF-1 [19]. MIF, however, competed with the recognized ligand for CXCR4 (SDF-1/CXCL12) for binding to CXCR4 [18].

CXCR4 is expressed by normal urothelium and may be associated with bladder cancer [20], [21]. Therefore, we hypothesized that CXCR4-MIF complex formation may also occur in the bladder (as described occurring in vitro [18]). Such associations, if present, would indicate another possible receptor target for MIF during cystitis, aside from the already described MIF-CD74 association [15].

The object of the present study was to determine if there was an association between MIF and CXCR4 receptors in the bladder. Therefore, we examined: 1) location of cytokine receptor CXCR4 in the rat bladder; 2) baseline bladder levels of SDF-1 (cognate ligand for CXCR4) and changes in response to a chemically-induced (cyclophosphamide; CYP) model of bladder inflammation; 3) CXCR4 expression changes after CYP-induced cystitis and 4) association between CXCR4 and MIF in the bladder before and after CYP-induced cystitis.

Our results show that both CXCR4 and SDF-1 are constitutively expressed in normal rat bladder and upregulated during CYP-induced cystitis. Using dual immunohistochemistry we show that MIF and CXCR4 are colocalized within the same cells in the urothelium and co-immunoprecipitation studies demonstrate MIF-CXCR4 associations in the bladder. These MIF-CXCR4 associations are increased during CYP-induced cystitis.

## Results

### Cyclophosphamide-induced bladder inflammation

Repeated measures ANOVA showed differences between saline- and CYP-treated rats in body weight, with significant decreases observed in CYP-treated rats as early as day 3 and continuing throughout the experiment (day 8; Table 1) but remaining below a 10% weight-loss threshold established as a protocol endpoint.

**Table 1:** Effect of cyclophosphamide on body weight (g)

Treatment	Day 0	Day 3	Day 6	Day 8
Saline (N = 10)	321±2.6 g	322±2.8	326±3.5	326±3.6
CYP (N = 10)	320±3.6	303±2.5***	300±3.0***	295±3.0***

Mean±S.E.M. Comparisons were made between Saline and CYP groups at each time point using post-hoc Bonferroni t-tests.

*** = p<0.001

**Figure 1:**
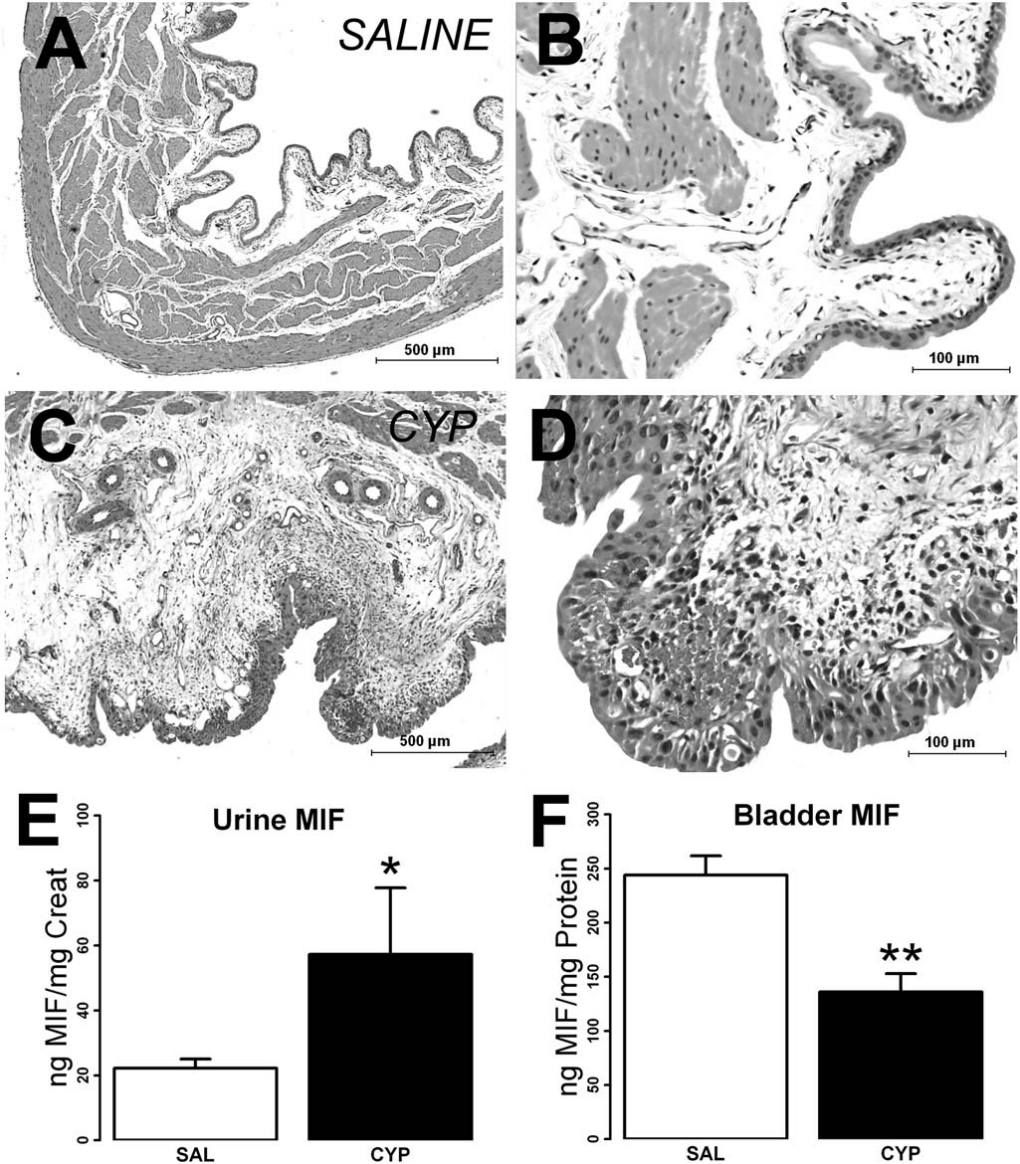
Effect of cyclophosphamide (CYP) treatment on bladder histology, bladder and urine MIF levels. Bladder paraffin sections stained with H&E showed normal morphology, as represented in A,B. CYP treatment however, caused significant edema and chronic inflammation (C,D), and some bladders had acute inflammatory cells. Asterisks in C,D mark areas of edema and high numbers of cellular infiltrates. Urothelial hyperplasia was also observed. In addition, urinary MIF levels were increased by CYP treatment (E; * = p<0.05), while bladder MIF levels were increased by CYP treatment (F; ** = p<0.01). Calibration bar: 1A,1C = 500 µm; 1B,1D = 100 µm.

In agreement with our previous findings in male Sprague-Dawley rats [6] , multiple CYP injections (40 mg/kg every third day for 8 days, a lower dose than reported effective for female Wistar rats; 75 mg/kg [22]) induced bladder inflammation. Hemorrhagic cystitis, however, was not observed in any of the CYP treated rats. Gross examination of the bladder under the dissecting microscope revealed lesions on the ventral luminal surface in all CYP-treated rats, but none of the saline-treated rats. H&E sections from saline treated rats showed normal bladder morphology (Figure 1A;B). CYP-treated rats, on the other hand, showed clear signs of inflammation, including, submucosal edema, disruption of urothelium, cellular infiltrates, hemorrhage and fibroblast proliferation (Figure 1C). While the urothelium in saline-treated rats showed the typical three-layer appearance (Fig 1B), CYP-treated rats showed hyperplasia (Fig. 1D).

Compared to saline treatment, CYP treatment increased urinary MIF levels (as measured by ELISA; Figure 1E; 22.2±2.8 vs. 57.3±20.5 in saline vs CYP, respectively; p<0.0464) while decreasing bladder MIF levels (Fig 1F; 244.1±39.7 vs 136.0±38.1 ng MIF/mg protein in saline vs CYP treatment respectively; p = .002), confirming our recent observations [6]. Circulating MIF represents a considerable source of MIF [9] and presents a possible confound when examining organ levels of MIF. The MIF ELISA developed in this study was not able to detect serum MIF. Therefore, our present results represent changes in tissue levels (or release) of MIF without the confounding presence of blood/serum MIF in the bladder or urine.

### CXCR4 immunostaining in urothelium: co-localization with MIF and effect of treatment with cyclophosphamide

**Figure 2:**
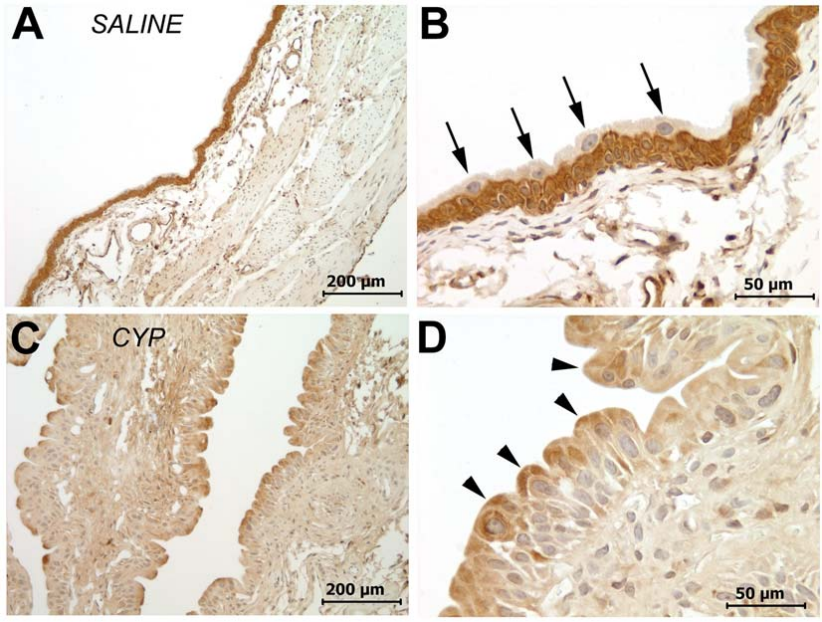
Representative CXCR4 immunostaining in urothelium of saline-treated (A;B) and CYP-treated (C,D) rats. Moderate to strong CXCR4 immunostaining was seen in basal and intermediate cells in the urothelium of saline treated rats (A;B), with little or no staining in superficial cells (B; arrows). In CYP-treated rats, there is a significant redistribution of CXCR4 immunostaining with decreased basal and intermediate cell staining (C;D) while superficial cells appeared stained and moderate staining in apical areas (D; arrowheads). Calibration bar: 2A,2C = 200 µm; 2B;2D = 50 µm.

Using standard immunoperoxidase protocols, moderate to strong CXCR4 immunostaining was observed in the urothelium of saline-treated rats, located in basal and intermediate layers, but not in superficial cells (Fig 2A,B). Following CYP-treatment, however, there was focal redistribution of CXCR4 immunostaining with superficial cells (and especially at their apical ends) showing moderate patchy staining (Fig 2C,D), while basal and intermediate cells appear to decrease in staining intensity. Overall urothelial staining intensity scores (as rated by blind observer) showed that cyclophosphamide-treated rats had a lower median score (1+0.75) compared to saline-treated rats (3+1; p = .0172; Wilcoxon rank sum test). Computerized analysis of immunostaining intensity showed that basal and intermediate cell staining decreased after CYP treatment (saline = 127.4+8.57; cyclophosphamide = 77.2+12.08; p = 0.0011), thus confirming redistribution of CXCR4 immunostaining.

**Figure 3:**
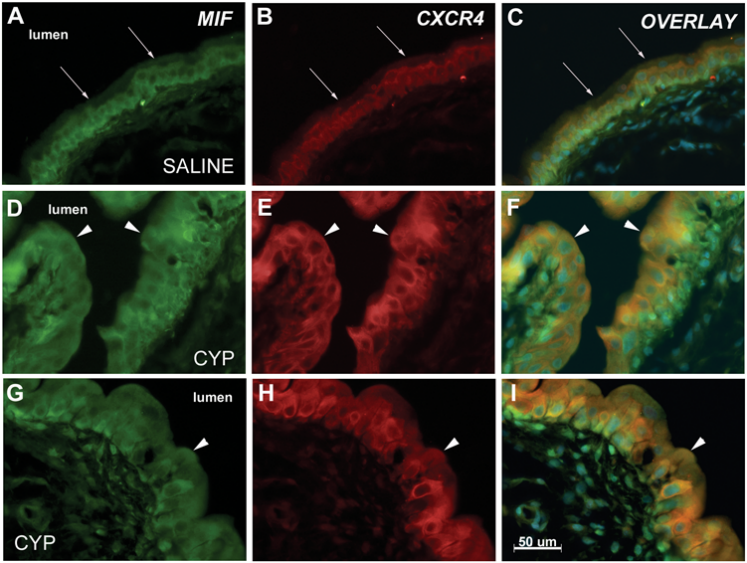
Co-localization of CXCR4 and MIF in urothelium. Representative sections from rats treated with saline (A–C) or CYP (D–I) are shown. The figure shows MIF immunostaining (green immunofluorescence), CXCR4 immunostaining (red immunofluorescence) and an overlay panel combining both immunostaining and a DAPI nuclear stain. MIF immunostaining is seen in basal and intermediate cells and in fibroblasts in the lamina propria of saline treated rats (A), while superficial cells do not stain for MIF. Arrows show luminal edge of urothelium. CXCR4 is restricted to basal and intermediate cells of urothelium (B) and lamina propria is not stained. Overlay of these panels (C) demonstrate co-localization of MIF and CXCR4 as orange coloring of cells. CYP treatment resulted in superficial cell staining for MIF (D,G) and CXCR4 (E,H) and overlay panels (F,I) demonstrate co-localization as orange color in urothelial cells. Arrows point to superficial cells showing MIF-CXCR4 co-localization. Calibration bar = 50 µm.

**Figure 4:**
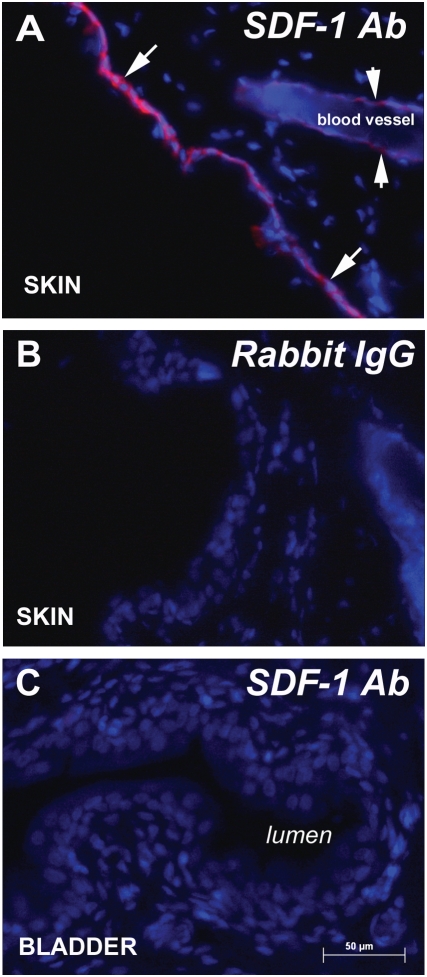
SDF-1 immunostaining. A) SDF-1 immunostaining was observed in keratinocytes (arrows) and endothelial cells in blood vessels (short arrows) in skin. B) Omission of primary antisera eliminated immunostaining. C) Bladder sections did not show SDF-1 immunostaining. Calibration bar = 50 µm.

We examined the co-localization of CXCR4 and MIF in the urothelium using dual-immunofluorescence. Figure 3 shows representative bladder sections from each group immunostained for MIF (FITC color), CXCR4 (TRITC color) and an overlay of those two panels (co-localization indicated by orange color; nuclear staining by DAPI shown in blue). In saline-treated rats, both MIF and CXCR4 could be localized in the basal and intermediate layers of the urothelium (but not in superficial cells) (Fig 4A–C). After CYP-treatment, MIF and CXCR4 are readily localized throughout the urothelium (Fig 4D–I), even on superficial cells previously devoid of MIF or CXCR4 (arrows in Fig 4F,I).

### Cyclosphosphamide increased bladder SDF-1 levels

We measured bladder levels of SDF-1, the cognate ligand for CXCR4, using ELISA. There was a significant difference between saline (4.4±1.0) and CYP-treated bladders (7.6±0.7 ng SDF-1/mg protein; p<0.05) in the levels of SDF-1. Spleen and skin were assayed as positive controls and showed the amount of SDF-1 in spleen is comparable to that found in the bladder (7.94 ng SDF-1/mg protein). Skin, on the other hand, had greater amounts of SDF-1 (16.04 ng SDF-1/mg protein) corresponding to approximately four and two times the amount of SDF-1 in saline and CYP-treated bladders, respectively. 

SDF-1 immunofluorescence was readily seen SDF-1 in skin keratinocytes and in endothelial cells in skin blood vessels (Fig 4A) as has been reported before [23]. However, SDF-1 immunostaining was not detected in bladder (Fig. 4C) or spleen (not shown).

### Cyclophosphamide upregulated bladder CXCR4 expression: MIF-CXCR4 associations in the bladder

**Figure 5:**
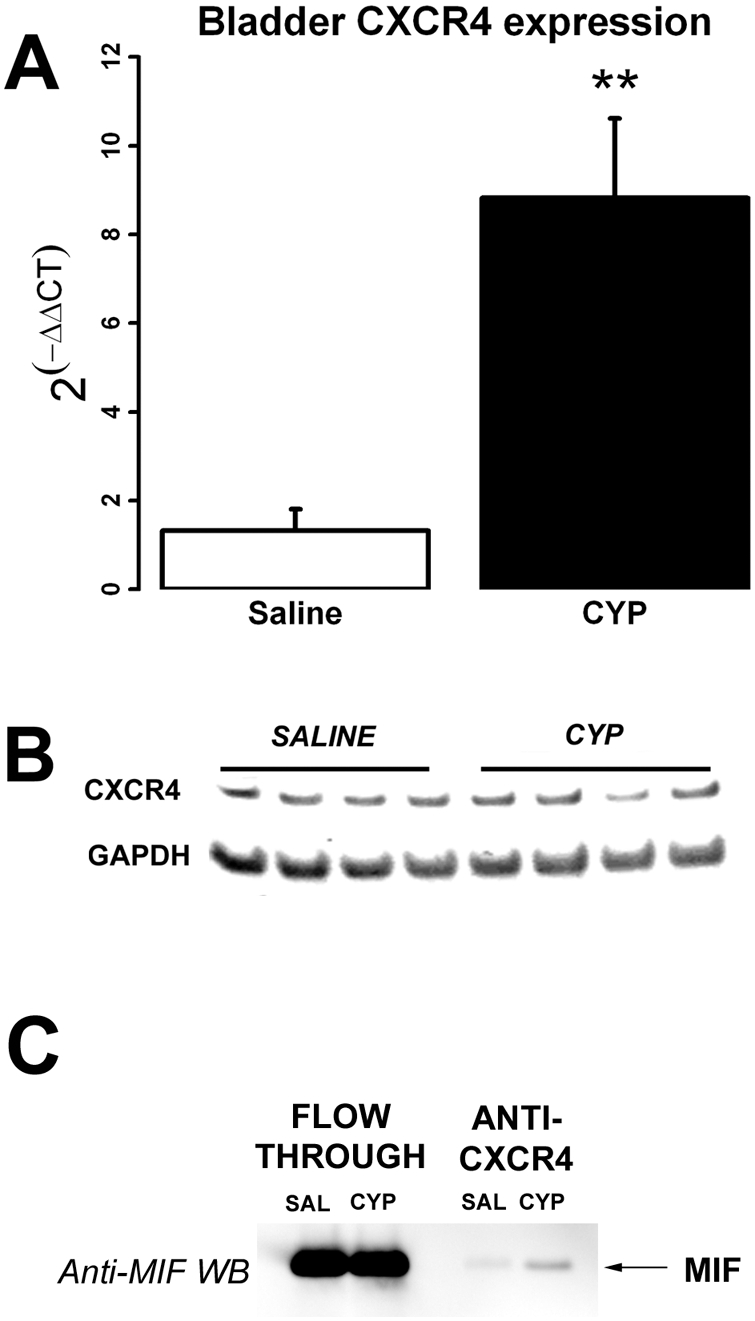
A) Real-time RT-PCR showed significant upregulation of bladder CXCR4 by CYP treatment (p = 0.004) compared to saline. B) CXCR4 Western-blotting showed no difference in CXCR4 amounts between saline and CYP-treated rats. GAPDH was used a loading control. C) Co-immunoprecipitation studies showed CXCR4-MIF associations in the bladder. A representative experiment is shown where MIF western-blotting of fractions from bladders of saline and CYP-treated rats were collected from the CXCR4 antibody column. CXCR4 antisera was used to “pull-down” CXCR4 complexes in bladder homogenates followed by MIF Western-blotting to detect CXCR4-MIF complexes. “Flow through” refers to fractions that did not adhere to the CXCR4 antibody column (and thus do not contain CXCR4-complexes), whereas “Anti-CXCR4” refers to fractions eluted from the CXCR4 antibody column (thus containing CXCR4 complexes). Most of the bladder MIF did not stick to the CXCR4 antibody column (“Flow-through”) and is not associated with CXCR4. However, a small amount was found co-immunoprecipitated with CXCR4 in saline-treated rats and these CXCR4-MIF complexes increased after CYP treatment (arrow). Densitometric analysis showed an increase of 3.5 fold in CXCR4-MIF complexes in CYP treated rats compared to saline controls.

Real-time PCR showed that CXCR4 mRNA was significantly upregulated in the bladder following CYP treatment (Figure 5A; ≈ 9-fold increase) when compared to saline. Western-blotting, on the other hand, showed equivalent levels of CXCR4 protein in the bladders of saline treated versus CYP-treated rats (Figure 5B).

Co-immunoprecipitation studies with CXCR4 antisera to “pull-down” CXCR4 protein complexes in bladder homogenates were followed by MIF Western-blotting in order to detect CXCR4-MIF complexes in bladder homogenates. Figure 5C shows a representative experiment where MIF western-blotting of fractions from bladders of saline and CYP-treated rats were collected from the CXCR4 antibody column. “Flow through” refers to fractions that did not adhere to the CXCR4 antibody column (and thus do not contain CXCR4-complexes), whereas “Anti-CXCR4” refers to fractions eluted from the CXCR4 antibody column (thus containing CXCR4 complexes). Note that most of the bladder MIF did not stick to the CXCR4 antibody column (“Flow-through”) and is not associated with CXCR4. However, a small amount was found co-immunoprecipitated with CXCR4 in the saline-treated rats and these CXCR4-MIF complexes increased after CYP treatment. Densitometric analysis showed an increase of 3.5 fold in CXCR4-MIF complexes in CYP treated rats compared to saline controls.

## Discussion

The results from the present study demonstrate that CXCR4, a chemokine cell-surface receptor, is constitutively expressed in normal rat urothelium localized to basal and intermediate cells. CYP treatment (aside from producing bladder inflammation and urothelial hyperplasia, well-described effects of cyclophosphamide in the bladder [24], [25]) also resulted in up-regulation of bladder CXCR4 mRNA and redistribution of CXCR4 to the entire urothelial area (including apical area of superficial cells previously devoid of CXCR4; Fig. 2D). Our findings of apical CXCR4 staining in superficial urothelial cells are in agreement with observations in colonic epithelial cells [26].

CYP treatment although producing CXCR4 mRNA upregulation (a novel finding) did not result in increased CXCR4 protein levels, and scoring of CXCR4 immunostaining actually showed a decrease in staining intensity following CYP treatment. A similar discrepancy between CXCR4 mRNA expression and protein levels has been reported in the rat neurons and shown to reflect activation, increased internalization and degradation of CXCR4 receptors [27] . Such activation, internalization and degradation of CXCR4 receptors may also account for the patchy CXCR4 immunostaining in the urothelium (especially with immunostaining in the apical surface of superficial cells) and may represent focal areas of CYP-induced CXCR4 response.

CXCR4 mRNA expression in normal human urothelium, bladder cancer and also bladder cancer cell lines (J82 and T24) was previously reported [20] . Addition of SDF-1 (presumably activating CXCR4 receptors) increased Matrigel invasion and cell growth but was not effective in increasing intracellular calcium in these particular urothelial cancer cells [20]. However, other investigators using a different bladder cell line (RT-4) did report an increase in intracellular calcium upon stimulation with SDF-1 [21]. Taken together these results suggest that CXCR4 receptors are functional in the urothelium. There is also evidence of CXCR4 mRNA expression in other areas of the human urogenital system (e.g. urethra, cervix) [28].

We examined protein levels of SDF-1 in the bladders of both saline-treated and CYP-treated rats using ELISA. We report constitutive levels of SDF-1 in saline-treated bladders which increase after CYP treatment. Although SDF-1 immunofluorescence was readily detectable in skin keratinocytes, we were unsuccessful in detecting SDF-1 by immunohistochemistry in the bladder (or the spleen). Since the levels of SDF-1 in the skin (measured by ELISA) are approximately twice the levels found in bladder or spleen, we consider it likely that the levels in these organs were below the detection level for immunohistochemistry.

Recently, down-regulation of SDF-1 mRNA expression in the bladder (and other pelvic viscera) was reported afer vaginal distension [29]. To our knowledge, our findings represent the first demonstration of SDF-1 protein levels in the bladder. Thus our findings indicate that bladder injury produced by CYP-treatment results in mRNA upregulation of the chemokine receptor CXCR4 and increased protein levels of its cognate ligand, SDF-1. In addition to several cytokines reported upregulated in the bladder after CYP treatment [30], changes in another chemokine (CX3CL1) and its receptor (CXC3R1) were described as a result of cyclophosphamide treatment [22]. Therefore, chemokines (in addition to cytokines) likely represent important mediators of bladder injury and possible targets for ameliorating bladder inflammation.

Until recently, CXCR4 was considered to bind exclusively to SDF-1 [19]. However, recent in vitro evidence showed that CXCR4 is also capable of binding MIF [18]. In this study we confirm these in vitro finding since we demonstrate 1) co-localization of CXCR4 and MIF in the urothelium, both in saline treated rats and after CYP treatment; 2) CXCR4-MIF associations are present in saline-treated bladder and increase after CYP treatment. Therefore, although, not directly tested in this study, our results suggest that MIF in the bladder may participate in bladder inflammation either through binding to CXCR4 in the urothelium (formerly thought to only bind SDF-1 but recently shown to also bind MIF [18]) or to CD74 (recognized binding protein for MIF which is upregulated in bladder inflammation [6]) to activate signal transduction pathways that result in the production of other inflammatory cytokines. Given that bladder MIF concentrations are approximately 30-fold greater than bladder SDF-1 concentrations, it is possible that MIF may be the primary ligand at the CXCR4 receptor in the urothelium. Moreover, CYP-treatment induced immunostaining of CXCR4 in superficial urothelial cells previously devoid of CXCR4 (or MIF) immunostaining. This raises the possibility that these cells will be activated by MIF present in the urine and in fact, CYP (present study) and other inflammatory stimuli [8]–[10] increase luminal MIF release. We cannot rule out a contribution of renal or ureteral MIF release to increased urine MIF levels observed in this study after CYP-treatment, Yet our current findings of increased urine MIF with concomitant decrease in bladder MIF protein levels are consistent with earlier findings where, in animals with bladders isolated from the kidneys (thus removing potential renal and ureteral contributions), we observed similar results [7], [9]). Thus, based on our experimental evidence we consider likely that MIF is released into the lumen from pre-formed stores in the bladder during inflammation. Therefore, luminal MIF may contribute to bladder inflammation through binding to at least these two urothelial cell-surface receptors and suggests that blocking MIF or cell-surface receptors associated with MIF may prevent or ameliorate bladder inflammation.

The exact role of CXCR4 and MIF-CXCR4 associations in the bladder was not addressed in the present study and remains to be investigated. However, recent evidence from other models suggests, at least, two interesting and important possibilities for the involvement of CXCR4 in bladder inflammation and repair from injury. First, CXCR4 may be mediating urothelial cell proliferation and repair. CXCR4 and SDF-1 are also expressed in human intestinal epithelial cells where recent evidence indicates it participates in epithelial repair following injury and maintaining mucosal barrier integrity [31], [32]. In this model then, chemokines and chemokine receptors (and particularly CXCR4/SDF-1) may represent an autocrine/paracrine loop that helps maintain mucosal barrier integrity and repair as well as regulating mucosal pathogenesis (including chronic inflammation as seen inflammatory bowel disease and progression to colon cancer) [33] . The results from the present study indicate that MIF, by associating with CXCR4 receptors in the bladder (presumably urothelial in origin), may be competing with SDF-1 at CXCR4 receptors to participate in epithelial repair following injury. Second, CXCR4 activation may mediate pain hypersensitivity in the bladder. Recent evidence has clearly shown expression of CXCR4 (and other chemokine receptors) on dorsal root ganglion (DRG) neurons [34]. In addition, activation of these receptors produced excitatory effects on DRG neurons and stimulated release of Substance P [34]. Also, CXCR4 (and other chemokine receptors and chemokines) expression was reported in DRG neurons following a rodent model of persistent neuropathy [35], [36]. These receptors were functional since intracellular calcium was increased following administration of SDF-1 in vitro [35]. Therefore, based on their findings, these authors suggest that chemokines and chemokines receptors may be important targets in the treatment of chronic pain. In CYP-induced cystitis, then, activation of CXCR4 receptors, either by its recognized ligand SDF-1 (upregulated as a result of CYP) or due to interaction with MIF (also upregulated as a result of CYP and present in greater quantities than SDF-1) may in fact be contributing to pain hypersensitivity. A similar suggestion has already been made for another chemokine, CX3CL1, and its receptor during CYP-induced cystitis [22]. Our results contribute to such a hypothesis and expand it to a different chemokine/receptor system.

In summary, the chemokine receptor CXCR4 is constitutively expressed in rat urothelium (in basal and intermediate cells) while CYP-induced bladder inflammation resulted in upregulation of CXCR4 and immunostaining of superficial cells (previously devoid of CXCR4). Its cognate chemokine ligand, SDF-1 is also upregulated by CYP-treatment. CXCR4 and MIF are co-localized in cells in the urothelium and CYP induced co-localization of CXCR4 and MIF to superficial cells of the urothelium. Immunoprecipitation demonstrated an association between CXCR4 and MIF in the bladder. Therefore, our results suggest that CXCR4, as a receptor for MIF in urothelial cells, may contribute to MIF-mediated bladder inflammation. Examining the differences between MIF activation of CXCR4 receptors versus MIF activation of CD74 receptors then might provide insights into the actual mechanism for MIF-mediated effects in the bladder and possibly other sites.

## Methods

All experiments were conducted after obtaining full institutional animal care and use committee approval and conformed to NIH Guide for animal experimentation. Bladder inflammation was produced using a recently published protocol whereby a reduced dose of cyclophosphamide (CYP) is administered to male Sprague-Dawley rats in order to avoid profound weight loss and mortality [6].

### Cyclophosphamide-induced cystitis

Male Sprague-Dawley rats (N = 20; 250–300 g; Harlan, IN, USA) were anesthetized with halothane and received either saline (vehicle control group; 0.1 ml/100 g body weight; i.p.; N = 10) or cyclophosphamide (CYP; Sigma, St Louis, MO, USA; in saline; 40 mg/kg; 0.1 ml/100 g body weight; i.p.; N = 10) every third day to induce cystitis [6]. Buprenorphine hydrochloride (0.03 mg/kg; s.c.; Reckitt Benckiser Pharmaceuticals Inc., Richmond, VA, USA) was also administered to each rat on the day of injection. Eight days after the first injection, ten rats (5 = saline; 5 = CYP) were anesthetized with sodium pentobarbital (60 mg/kg; i.p.; Ovation Pharmaceuticals, Deerfield, IL, USA) and perfused with saline followed by 4% paraformaldehyde and the bladders collected for histology. Alternatively, rats were re-anesthetized with halothane (N = 10; 5 = saline; 5 = CYP-treated), bladders were exposed through an abdominal incision and urine collected (using a syringe with a 32 gauge needle). Bladders were excised and quickly frozen (−80°C) for protein or mRNA extraction and the rats were euthanatized.

### Histology and Immunohistochemistry

Formaldehyde-fixed bladders were cut coronally through the mid-detrusor region and embedded in paraffin. Paraffin sections (4 µm) were stained with hematoxylin and eosin or processed for CXCR4 immunohistochemistry as follows: Slides were deparaffinized and subjected to antigen retrieval using citrate buffer (pH 6.0; 95°C for 30 min). Endogenous peroxide was blocked by incubating the slides in 3% H_2_O_2_ for 3 min. The section were exposed to CXCR4 antisera (1∶2000; rabbit-polyclonal; Sigma; #C3116) overnight at 4°C and then processed using a standard ABC reaction according to the manufacturer's protocol (IHC Select; Chemicon, Temecula, CA, USA). Sections were lightly counterstained with hematoxylin, coverslipped and examined using a Leica inverted microscope. Immunostaining intensity was rated by a pathologist (KAI) blind to experimental conditions from 0 (no staining) to 4 (strong immunostaining). In addition, digital images were analyzed for CXCR4 immunostaning intensity using Image J (NIH; Bethesda, MD) and proprietary custom written plug-ins (University of Colorado-Denver; Prostate Diagnostic Laboratories, Denver, Aurora).

Frozen (coronal) bladder sections (12 µm) of mid-detrusor were exposed simultaneously to both MIF (1∶200; goat-polyclonal; Novus Biological, Littleton, CO, USA; #NB100-1789) and CXCR4 antisera (1∶200; rabbit-polyclonal; Sigma; #C3116). The ability of this MIF antibody to recognize MIF was verified by preliminary western-blots using rat recombinant MIF (gift from Torrey Pines) and rat tissue homogenates (data not shown). Primary antisera were visualized using appropriate secondary antisera conjugated to fluorescein isothiocyanate (FITC; Jackson Immunochemicals; West Grove, PA, USA; #705-095-147) or tetramethylrhodamine isothiocyanate (TRITC; Jackson Immunochemicals; #711-025-152). Sections were coverslipped with fade-retardant medium (Prolong Gold with DAPI; Invitrogen; Carlsbad, CA, USA) and examined using a Leica (Leica microsystems, Wetzlar, Germany) inverted microscope, equipped with a Leica digital camera. Overlay pictures showing MIF (green; FITC), CXCR4 (red; TRITC) and DAPI nuclear counterstaining were obtained with Leica software. Control sections included omission of either or both primariy antisera, or omission of either or both secondary antisera.

For SDF-1 immunohistochemistry, frozen bladder sections were exposed to SDF-1 antisera (rabbit polyclonal; Torrey Pines Biolabs, Houston, TX, USA; TP201;1∶200; overnight 4°C) and visualized with TRITC. Spleen and skin sections were used as positive controls.

### MIF and SDF-1 ELISA

Urine and bladders from saline and CYP-treated rats were assayed for levels of MIF and SDF-1 (bladders only) using enzyme-linked immunoabsorbent assay (ELISA). MIF ELISA was also tested for its ability to detect serum MIF. Briefly, high binding ELISA plates (Microlite 2, ThermoScientific, Waltham, MA, USA) were coated with 100 µl of primary antibody (1 µg/ml; anti-MIF; Abcam; Cambridge, MA, USA; #ab7207 or anti-SDF-1; Torrey Pines Biolabs; TP201) at room temperature overnight. The ability of this MIF antibody to recognize MIF was verified by preliminary western-blots using rat recombinant MIF (gift from Torrey Pines) and rat tissue homogenates (data not shown). Plates were blocked with 200 µl reagent diluent (1% BSA in PBS pH 7.4) for 1 h at room temperature. Recombinant rat MIF (a gift from Torrey Pines) or recombinant SDF-1 (Preprotech Inc; Rocky Hill, NJ, USA) was used to generate a standard curve from 1.6 to 100 ng/ml in reagent diluent. Samples were diluted to the appropriate concentration in reagent diluent and applied in duplicate wells. Plates were covered with adhesive tape and incubated 2 h at room temperature. Individual wells were then washed three times with wash buffer (PBS containing 0.05% Tween-20, pH 7.4) using an automated plate washer (ThermoScientific). Detection antibody (biotinylated-goat anti-MIF, BAF289, R&D Systems, Minneapolis, MN, USA or biotinylated goat anti-SDF-1, R&D Systems) was added at a final concentration of 200 ng/ml, the plates recovered with adhesive and incubated 2 h at room temperature. The wells were washed as described above, 100 µl streptavidin-horseradish peroxidase (1∶200 dilution in reagent diluent, DY998, R&D Systems) added to each well and the covered plate was incubated for 20 minutes at room temperature. The wells were washed as described above, 100 µl peroxidase substrate (R&D; DY999) was added and each well read using a plate reader (Biotek, Winooski, VT, USA). A standard curve was created using linear regression and sample concentrations calculated by interpolation (PRISM 4.02, GraphPad Software, La Jolla, CA, USA). Assays had an inter- and intra-plate coefficient of variation of 15.3% and 18%, respectively for MIF and 7.2% (both for inter- and intraplate) for SDF-1. Bladder MIF and SDF-1 levels are expressed normalized to protein amount (BCA, Thermo Scientific) present in the samples. Urine creatinine was determined using a commercially available assay (Exocell, Philadelphia, PA, USA) and performed according to the manufacturer's protocol. Urine MIF levels are normalized to urine creatinine levels in the samples.

### Bladder CXCR4 mRNA expression, Western blotting and CXCR4-MIF co-immunoprecipitation

Total RNA was isolated from bladder tissues using Trizol (Invitrogen) and CXCR4 (SuperArray primer; PPR06440A; SABiosciences, Frederick, MD, USA) and 18S rRNA (control; SuperArray: PPR57734E) gene expression was determined by Real-time RT-PCR (Opticon, Bio-Rad, Hercula, CA, USA) using SYBR Green incorporation and the ΔΔCT method of analysis. Differences in bladder CXCR4 mRNA expression between saline-treated and cyclosphamide-treated rats were determined using a Student's t-test. p<0.05 was considered significant.

Western-blotting of bladder homogenates was performed under non-reducing conditions following the manufacturer's protocols (NuPAGE Bis-Tris gels, Invitrogen) as described previously [8]. Briefly, 20 µl of bladder homogenates were loaded onto NuPAGE Bis-Tris gels (4–12%; Invitrogen). After electrophoresis, separated proteins were transferred to a polyvinylidene fluoride membrane. CXCR4 protein bands were detected using a polyclonal antibody to CXCR4 (Sigma; C3116) and chemiluminescent substrate (Sigma 1∶1000). Glyceraldehyde-3-phosphate dehydrogenase (GAPDH) was used as a loading control. Band intensities were quantified using Kodak Image Station (Kodak, Rochester, NY, USA) and expressed as a ratio of the saline group.

In order to detect MIF binding to CXCR4 in the bladder, we used CXCR4 co-immunoprecipitation followed by MIF western blotting. CXCR4 was precipitated from frozen bladder tissue homogenates (200 µg of total protein) using CXCR4 antibody (5 µg, Sigma; #C3116). CXCR4 containing protein complexes were isolated using Protein G agarose beads and then separated by denaturing, reducing SDS electrophoresis. MIF containing bands were identified by Western blotting using biotinylated anti-MIF antibody (1∶1000 dilution, R&D Systems).

### Statistical Analysis

Generally, data are presented as Mean±S.E.M and group differences were determined with t-tests. Visual scoring of CXCR4 immunostaining is presented as Median±Interquartile range and differences between the two groups were assessed using a Wilcoxon rank sum test, whereas digitized CXCR4 immunostaining are presented as Mean±S.E.M of arbitrary intensity scores and differerences are analyzed using t-tests. Body weight loss as a function of cyclophosphamide treatment was tested using two-way (Treatment x Time) repeated-measures analysis of variance (ANOVA). Bonferroni post-hoc t-tests examined body weight differences between saline and cyclophosphamide groups at specific time points.. Analyses were conducted using statistical software (R; http://www.r-project.org/).
